# SARS-CoV-2 Genome Sequencing Methods Differ in Their Abilities To Detect Variants from Low-Viral-Load Samples

**DOI:** 10.1128/JCM.01046-21

**Published:** 2021-10-19

**Authors:** C. Lam, K. Gray, M. Gall, R. Sadsad, A. Arnott, J. Johnson-Mackinnon, W. Fong, K. Basile, J. Kok, D. E. Dwyer, V. Sintchenko, R. J. Rockett

**Affiliations:** a Centre for Infectious Diseases and Microbiology-Public Health, Westmead Hospital, Westmead, New South Wales, Australia; b Centre for Infectious Diseases and Microbiology Laboratory Services, NSW Health Pathology, Institute for Clinical Pathology and Medical Research, Westmead, New South Wales, Australia; c Sydney Medical School, University of Sydney, Sydney, New South Wales, Australia; d Sydney Informatics Hub, Core Research Facility, University of Sydney, Sydney, New South Wales, Australia; National Institute of Allergy and Infectious Diseases

**Keywords:** genomics, SARS-CoV-2, public health, variants

## Abstract

Severe acute respiratory syndrome coronavirus 2 (SARS-CoV-2) genomic surveillance has been vital in understanding the spread of coronavirus disease 2019 (COVID-19), the emergence of viral escape mutants, and variants of concern. However, low viral loads in clinical specimens affect variant calling for phylogenetic analyses and detection of low-frequency variants, important in uncovering infection transmission chains. We systematically evaluated three widely adopted SARS-CoV-2 whole-genome sequencing methods for their sensitivity, specificity, and ability to reliably detect low-frequency variants. Our analyses reveal that the ARTIC v3 protocol consistently displays high sensitivity for generating complete genomes at low viral loads compared with the probe-based Illumina Respiratory Viral Oligo panel and a pooled long-amplicon method. We show substantial variability in the number and location of low-frequency variants detected using the three methods, highlighting the importance of selecting appropriate methods to obtain high-quality sequence data from low-viral-load samples for public health and genomic surveillance purposes.

## INTRODUCTION

The rapid implementation of genomic epidemiology has enabled unparalleled understanding and monitoring of viral evolution during the severe acute respiratory syndrome coronavirus 2 (SARS-CoV-2) pandemic. The first report of a SARS-CoV-2 case in Australia was on 25 January 2020, and by the end of May 2021, 30,106 SARS-CoV-2 cases had been identified nationwide (https://www.health.gov.au/resources/publications/coronavirus-covid-19-at-a-glance-31-may-2021). Australia’s low prevalence of coronavirus disease 2019 (COVID-19) is due to the implementation of strong public health measures, which in New South Wales (NSW) has included integrated genomic surveillance to inform public health responses and contact tracing efforts (1).

Whole-genome sequencing (WGS) of SARS-CoV-2 was implemented in NSW within 2 weeks of the first reported case in anticipation of increasing SARS-CoV-2 infections (2). A pooled long-amplicon (long-amp)-based sequencing approach was initially selected based on reagent and resource availability and was quickly adapted to fit existing WGS workflows and infrastructure (3). By 28 March 2020, 209 samples from NSW had been sequenced and released on the Global Initiative on Sharing All Influenza Data database (GISAID; www.gisaid.org) (3), representing 13% of all SARS-CoV-2 cases diagnosed in NSW at the time. The initiative to promptly release genomic data has mirrored other national and international efforts focused on near real-time monitoring of the evolution and intercontinental spread of the SARS-CoV-2 (4–6). Prospective WGS of SARS-CoV-2 cases in NSW has continued, and to date (30 June 2021), 1,865 genomes representing 34% of confirmed cases have been generated.

An array of SARS-CoV-2 sequencing protocols have been developed since the start of the pandemic. The most commonly used methods contain numerous primers or baits which select and enrich overlapping fragments of the SARS-CoV-2 genome directly from clinical samples. This amplification or enrichment step is required, as products of pure metagenomic approaches are dominated by host nucleic acid molecules, which are several orders of magnitude larger than the SARS-CoV-2 genome. Even after SARS-CoV-2 enrichment/amplification, most high-throughput sequencing methods require a significantly higher input viral load than molecular diagnostics assays, limiting the number of SARS-CoV-2 genomes that can be generated from low SARS-CoV-2 yield samples.

In addition to wet laboratory techniques, a suite of bioinformatics and data visualization workflows have been developed, enabling global comparisons of SARS-CoV-2 genomes (7). The rapid development of all aspects of SARS-CoV-2 WGS was aided in part by efforts from the global genomics community in developing viral WGS methods (https://artic.network/ncov-2019). However, accurate SARS-CoV-2 genomic surveillance has been hampered by several common challenges. First, a high level of variability exists between sequencing protocols in obtaining complete SARS-CoV-2 genomes, particularly from clinical samples with low viral loads (as reflected by real-time PCR [RT-PCR] cycle threshold [*C_T_*] values), such as those collected from patients without symptoms, with mild disease, or late in the course of infection. Second, the accuracy required to detect and call variants using different protocols has not been adequately validated. All of these factors—sequencing method, reproducibility, and thresholds for variant calling—may affect the quality and impact of genomic surveillance and ultimately public health efforts to contain outbreaks.

Synthesis of SARS-CoV-2 genomic data with detailed epidemiological exposure and contact tracing information can provide definitive evidence of importation events and identification of local SARS-CoV-2 transmission chains (3, 8). SARS-CoV-2 clusters, transmission chains, or networks linked to superspreading events are often differentiated genomically by single nucleotide polymorphisms (SNPs) within the SARS-CoV-2 genome (9). The ability to rapidly and accurately characterize SNPs and other variants has become even more important after the identification of several so-called variants of concern (VOC). VOC contain specific mutations identified as important and relevant for COVID-19 control due to mounting evidence of positive selection of specific nonsynonymous spike protein mutations that can increase the duration, severity, and transmission of COVID-19 by affecting host immune responses (10–14). Complete genomes generated using highly sensitive and specific sequencing methods are therefore required to inform and enable genomics-guided surveillance to provide the information necessary for COVID-19 control and policy decisions, particularly as widespread SARS-CoV-2 vaccination is under way (15–17).

This study systematically evaluated three different sequencing methods for their sensitivity and ability to generate complete SARS-CoV-2 genome sequences suitable for public health surveillance. We assessed and compared (i) the pooled long-amplicon (long-amp) method (2) with (ii) the ARTIC v3 network tiled amplicon protocol (https://artic.network/ncov-2019), which has been adopted widely since the start of the pandemic, and (iii) a probe capture-based panel, the Respiratory Viral Oligo panel (RVOP) (Illumina). Additionally, we investigated the pattern of low-frequency variants generated by these methods, which can be important in defining and highlighting transmission chains (18–20).

## MATERIALS AND METHODS

### Clinical specimens.

The study period and region included the 4 months between March and July 2020 in NSW, Australia. SARS-CoV-2 RT-PCR-positive specimens which were subsequently cultured at NSW Health Pathology–Institute of Clinical Pathology and Medical Research (ICPMR) in the study period were included for selection. Respiratory samples in universal transport medium (UTM) which were RT-PCR negative for SARS-CoV-2 were collected and stored at 4°C. These negative specimens were deidentified and pooled, totaling 40 ml, before RNA was extracted. This RNA was used to dilute SARS-CoV-2 isolates, referred to here as negative respiratory matrix. Ethical and governance approval for the study was granted by the Western Sydney Local Health District Human Research Ethics Committee (2020/ETH02426).

### Viral isolation.

SARS-CoV-2-positive respiratory specimens were cultured in Vero C1008 cells (Vero 76, clone E6, Vero E6 [ECACC 85020206]) as previously outlined (21). Briefly, Vero cell cultures were seeded at 1 × 10^4^ to 3 × 10^4^ cells/cm^2^ in Dulbecco’s minimal essential medium (DMEM; Lonza, Alpharetta, GA, USA) supplemented with 9% fetal bovine serum (FBS; HyClone, Cytiva, Sydney, Australia) in 25-cm^2^ cell culture flasks (Corning, NY). Medium was replaced within 12 h with inoculation medium containing 1% FBS with the addition of penicillin, streptomycin, and amphotericin B deoxycholate to prevent microbial overgrowth and then inoculated with 500 μl of SARS-CoV-2-positive respiratory sample. The inoculated cultures were incubated at 37°C in 5% CO_2_ for 5 days (days 0 to 4). Cell cultures were observed daily for cytopathic effect (CPE). Routine mycoplasma testing was performed to exclude mycoplasma contamination of the cell line, and all culture work was undertaken in physical containment laboratory level 3 (PC3) biosafety conditions. The presence of CPE and increasing viral load was indicative of positive SARS-CoV-2 isolation. RT-PCR testing was performed on day 1, 2, 3, and 4 by conducting RNA extraction and SARS-CoV-2 RT-PCR on 200 μl of culture supernatant. Culture supernatant was harvested 4 days after inoculation and stored at −80°C.

### RNA extraction from viral culture.

A total of 600 μl (three 200-μl portions) of day 4 SARS-CoV-2 culture supernatant was used as input into the RNeasy minikit (Qiagen) for RNA extraction with minor modifications. Six hundred microliters of RNeasy lysis buffer was added to 200 μl of sample and mixed well. An equal volume (800 μl) of 70% ethanol was then added and mixed well by pipetting, before loading onto RNeasy column in successive aliquots until the entire volume was extracted. RNA was eluted in 30 μl, pooled for a total of 90 μl, and stored at −80°C prior to dilution. Total RNA was extracted from pooled SARS-CoV-2-negative clinical specimens as described above.

### Respiratory virus detection by RT-PCR.

A previously described RT-PCR (22) targeting the N gene was employed to estimate the viral load of cultured RNA and ensure the absence of SARS-CoV-2 in the negative respiratory matrix. Additional RT-PCRs were used to investigate the presence of common viral respiratory viruses: human influenza viruses A and B, parainfluenzaviruses 1, 2, and 3, respiratory syncytial virus, adenovirus, and rhinovirus in negative UTM extract (23).

### Synthetic control.

A commercially available synthetic RNA control reference strain (Wuhan-1 strain; TWIST Biosciences) containing six nonoverlapping fragments replicating the most commonly used reference sequence (NCBI GenBank accession no. MN908947.3) was used as a control to monitor SNPs and low-frequency variants that are artifacts of the viral amplification or sequencing process. The genomic coordinates of five nonoverlapping segments were not available from the manufacturer; therefore, we were unable to determine the genomic segments that may be affected by the noncontiguous fragments prior to library preparation and sequencing. Serial 10-fold dilutions from 20,000 copies/μl to 2 copies/μl were made and used to generate a standard curve and quantify the viral load of each culture spiked dilution per reaction. N gene SARS-CoV-2 RT-PCR was used to determine the viral load of the neat culture RNA after extraction. The synthetic control was also serially diluted 10-fold in respiratory matrix (as outlined below), enriched using each of the methods described below and sequenced in parallel with diluted cultures.

### Normalization and serial dilution of viral culture RNA into negative respiratory matrix.

Based on the viral load of the neat culture RNA (*C_T_*, 12.57 to 14.48; viral load, 2.0 × 10^8^ to 6.0 × 10^7^ copies/μl), each culture RNA extract was diluted 1:10 with negative RNA extract. Then 10-fold serial dilutions were made in negative RNA extract until an estimated concentration of >10 copies/μl (*C_T_*, 37 to 40) was reached for each isolate. cDNA was generated for all serially diluted RNA samples using a LunaScript RT SuperMix kit (New England BioLabs). Sufficient volume was prepared to perform duplicates for each method at each dilution. RNA and corresponding cDNA dilutions were aliquoted and stored at −80°C and −20°C, respectively. RT-PCR was then performed for each sample dilution to determine *C_T_* value and corresponding viral load.

### Viral enrichment and genome sequencing.

For each of the serially diluted samples, viral enrichment was performed using three methods: ARTIC v3, a 14-pool long-amplicon (long-amp) approach, and probe capture using Illumina RNA preparation with enrichment with the Respiratory Viral Oligo panel (RVOP). Resulting libraries were pooled with the aim of generating 1 × 10^6^ raw reads per specimen. Details of each enrichment method are outlined below.

### (i) ARTIC v3 nCoV-2019 sequencing protocol.

The ARTIC v3 protocol (https://www.protocols.io/view/ncov-2019-sequencing-protocol-v3-locost-bh42j8ye) was performed with the following modifications. Tiling PCR was used to amplify the whole genome according to ARTIC nCoV2-2019 sequencing protocol. Each PCR included 12.5 μl Q5 high-fidelity 2× master mix (New England Biolabs), 3.6 μl of either pool 1 or pool 2 10 μM primer master mix (final concentration of each primer was ∼10 to 11 pM), and 5 μl of template; molecular-grade water was added to generate a total volume of 25 μl. Cycling conditions were as follows: initial denaturation at 95°C for 2 min, followed by 35 cycles of 95°C for 30 s and 63°C for 2 min 45 s, and a final extension step of 75°C for 10 min. Pool 1 and pool 2 amplicons were combined, purified with a 1:1 ratio of AMPure XP beads (Beckman Coulter), and eluted in 30 μl of sterile water. Purified products were quantified using Qubit 1× double-stranded-DNA (dsDNA) high-sensitivity (HS) assay kit (Thermo Fisher Scientific) and diluted to the desired input concentration for library preparation. Sequencing libraries were prepared using Nextera XT (Illumina) according to manufacturers’ respective instructions. Sequencing libraries were then sequenced as 2 × 150-bp reads on either the Illumina iSeq or MiniSeq platform.

An updated ARTIC v3 protocol with rebalanced primer pools was also evaluated in this study. Primers for each ARTIC v3 pool were combined according to updated COG-UK consortium guidelines (https://www.protocols.io/view/covid-19-artic-v3-illumina-library-construction-an-bky5kxy6). Subsequent PCR and sequencing using the rebalanced ARTIC primer pools were performed as described above.

### (ii) Pooled long-amplicon PCR.

Pooled long-amplicon sequencing (dx.doi.org/10.17504/protocols.io.befyjbpw) was performed as described previously (2). Briefly, 14 overlapping PCR amplicons were independently generated and pooled in equal volumes. Pooled products were purified with 0.8× AMPure XP beads (Beckman Coulter) and eluted in 30 μl of sterile water. Qubit 1× dsDNA HS assay kit (Thermo Fisher Scientific) was used to quantify pooled amplicons before diluting to the desired input concentration for library preparation. Sequencing libraries were prepared using the Nextera XT kit (Illumina) and sequenced on either iSeq or MiniSeq (Illumina) using 2 × 76-bp paired-end reads. No other changes were made to the protocol.

### (iii) Respiratory Viral Oligo panel.

Diluted culture RNA extracts were used as input into the RNA Prep with Enrichment kit (Illumina). RNA denaturation, first- and second-strand cDNA synthesis, cDNA tagmentation, library construction, cleanup, and normalization were performed according to manufacturer’s instructions. Individual libraries were then combined in 3-plex reactions for probe hybridization. The Respiratory Viral Oligo panel v2 (Illumina) was used for probe hybridization with the final hybridization step held at 58°C overnight. Hybridized probes were then captured and washed according to manufacturer’s instructions and amplified as follows: initial denaturation 98°C for 30 s, 14 cycles of: 98°C for 10 s, 60°C for 30 s, 72°C for 30 s, and a final 72°C for 5 min. Library quantities and fragment size were determined using a Qubit 1× dsDNA HS assay and Agilent HS Tapestation and sequenced using 2 × 76-bp runs on the Illumina MiniSeq. using BWA-mem version 0.7.17. SAMtools v1.10 was used to curate BAM files with an average mapping quality threshold (MAPQ) of ≥60 and an average read depth of ≥10 and to calculate average genome coverage for each reference sequence.

### Bioinformatic analysis.

Raw sequence data were processed using an in-house quality control procedure prior to further analysis. Demultiplexed reads were quality trimmed using Trimmomatic v0.36 (sliding window of 4, minimum read quality score of 20, leading/trailing quality of 5, and minimum length of 36 after trimming) (24). Reference mapping and variant calling was performed using iVar version 1.2 (25). Briefly, reads were mapped to the reference SARS-CoV-2 genome (NCBI GenBank accession no. MN908947.3) using BWA-mem version 0.7.17, with unmapped reads discarded. Primer positions were supplied to iVar trim to soft-clip any reads in the bam file which matched primer sequences. Average genome coverage was estimated by determining the number of missing bases (Ns) in each sequenced genome. Variants were called using iVar variants (minimum read depth, >10×; quality, >20; minimum frequency threshold, 0.1). SNPs were defined based on an alternative frequency of ≥0.9, whereas low-frequency variants were defined by an alternative frequency between 0.1 and 0.9. Low-frequency variants with <100× depth were excluded over concerns over reliability of calls where the frequency of either allele dropped below 10. Low-frequency variants were included only if they were detected in 2 or more dilutions of each spike culture sequenced. Variants falling in the 5′ and 3′ untranslated regions were excluded due to poor sequencing quality of these regions. Polymorphic sites that have previously been highlighted as problematic were monitored (26). SARS-CoV-2 lineages were inferred using Phylogenetic Assignment of Named Global Outbreak LINeages v2 (PANGOLIN) (https://github.com/hCoV-2019/pangolin) (27). The frequency and positions of polymorphisms were compared between dilutions of the same culture and also against the original genome generated from the respiratory specimen and between cultures. Median genome coverage was calculated using the median depth in 50-bp bins across the reference genome for each method and dilution. Median read depth per amplicon was assessed in nonoverlapping segments of each ARTIC v3 amplicon, which was then converted to a factor of the expected read coverage (total mapped reads/genome size × 150 bp). These factors were compared between original and rebalanced ARTIC v3 sequencing runs. To detect other respiratory pathogens using RVOP, quality control (QC)-processed and trimmed reads from diluted cultures prepared using the RVOP were mapped against 203 reference sequences of 43 respiratory pathogens using BWA-mem version 0.7.17. SAMtools v1.10 was used to curate BAM files with an average MAPQ of ≥60 and an average read depth of ≥10 and to calculate average genome coverage for each reference sequence. Manual inspection of BAM files was conducted to confirm pathogen detection. Graphs were generated using R (version 3.6.1).

### Analytical performance: sensitivity and specificity.

Sensitivity and specificity were calculated for each sequencing method using a consensus SNP approach. For each isolate, a SNP called in any method was considered a true-positive SNP if it occurred in two or more sequencing methods at the highest dilution. SNPs identified by a single sequencing method only (and not detected in the original clinical specimen) were considered false positives. Sensitivity was calculated using the formula *A*/(*A* + *C*) × 100, where *A* is the number of true-positive SNPs and *C* is the number of false-negative SNPs. Specificity was calculated using the formula *D*/(*D* + *B*) × 100, where *D* is the number of true-negative bases (within the coding sequence [CDS] region) and *B* is the number of false-positive SNPs. Pairwise statistical comparisons were conducted between genome coverage and sensitivities at each dilution across each method using the Friedman test or Mann-Whitney tests with a significance level at a *P* value <0.05.

### Cost and turnaround time.

The hands-on-time and sequencing turnaround time were calculated for each method described. An estimation of the cost (in Australian dollars) of each method was also conducted. The costing takes into account all laboratory consumables but excludes labor (see Table S5 in the supplemental material [supplemental file 5]).

### Data availability.

Fastq files have been deposited in BioProject under accession no. PRJNA723901 for all 118 genomes produced in this study. Individual SRA and GISAID accessions and tabulated details of raw and mapped reads can be found in Table S4 (supplemental file 7) and Fig. S1 (supplemental files 1 and 6), respectively.

## RESULTS

### Viral isolates, viral loads, and genome profiles.

**FIG 1:**
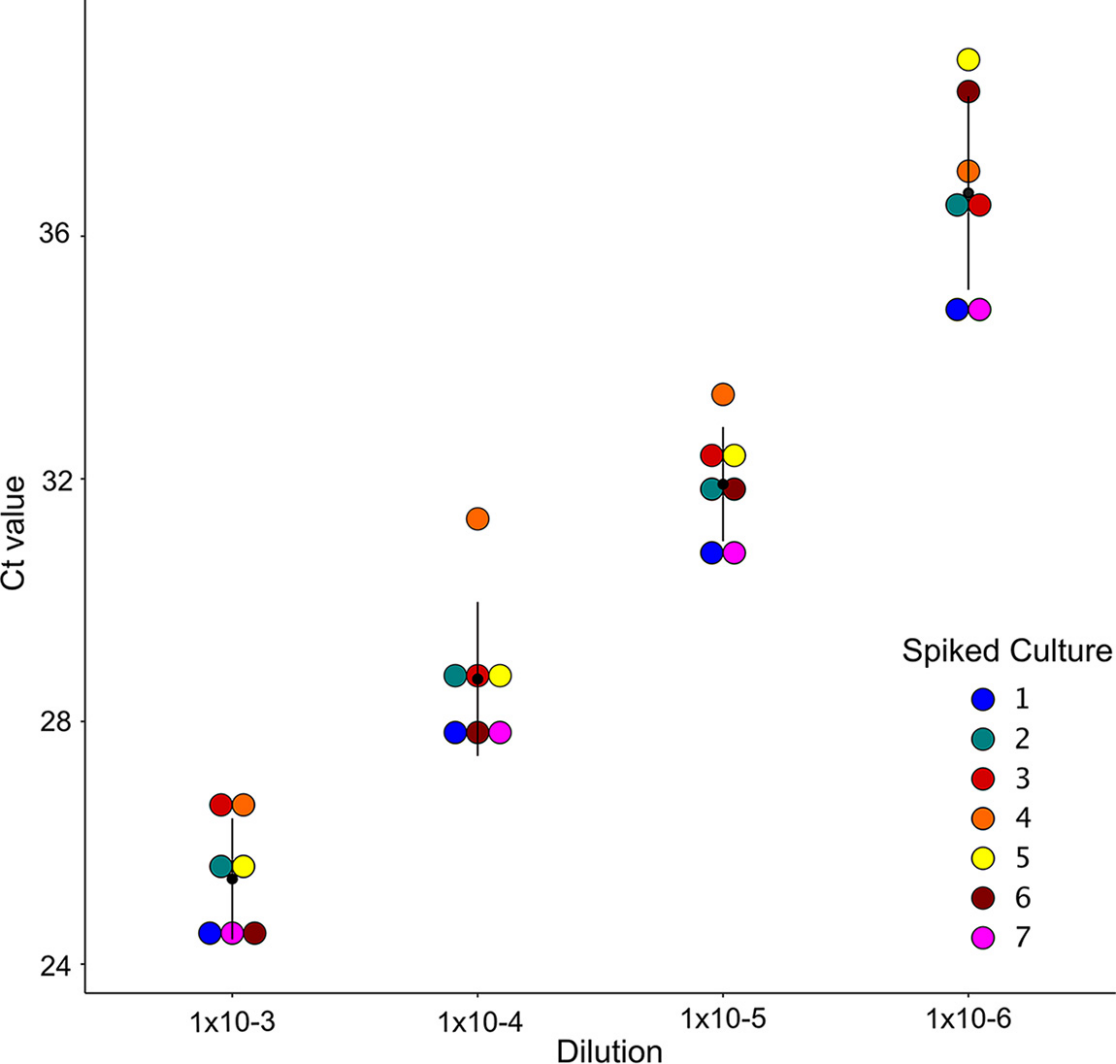
Viral load of SARS-CoV-2 cultures spiked in respiratory matrix. RT-PCR quantification of seven serially diluted SARS-CoV-2 cultures demonstrates an increase of 3 to 4 cycles for each 10-fold dilution of viral culture. The black dot represents the median *C_T_* value at each dilution, and the black lines represent the interquartile range.

Seven SARS-CoV-2-positive clinical specimens were cultured as representatives of different SARS-CoV-2 genomic clusters that were cocirculating in NSW between February and April 2020 (3). Details of the genome obtained from each clinical specimen, including GISAID ID, lineage, and SNP profile, are listed in Table S1 (supplemental file 5). Two of seven isolates lost a SNP compared to the genome obtained directly from the original clinical specimens. The genome of isolate 2 reverted to wild type at position C:26213; however, the SNP C:26213:T detected in the original clinical specimen was still present as a low-frequency variant. In isolate 7, all reads at position 13730 were the wild-type allele (C). To investigate the effect of low viral load on detection of variants, serial dilutions of cell culture supernatant were performed. RT-PCR results from each culture dilution demonstrated that a 10-fold decrease in viral load corresponded to a *C_T_* increase of ∼3 to 4 cycles (Fig. 1). A total of seven dilutions were made, five of which remained consistently SARS-CoV-2 RT-PCR positive, with corresponding viral loads decreasing from a median of 71,062 copies/μl (median *C_T_*, 25.42; range, 24.29 to 26.65; viral load range, 47,482 to 1,178,540 copies/μl) to a median of 112 copies/μl (median *C_T_*, 36.62; range, 34.7 to 38.19; viral load range, 18 to 1,584 copies/μl). Culture dilutions with *C_T_* values of >39 were deemed too low to attempt sequencing and were excluded from further analysis.

### Synthetic control.

Using the long-amp method, only 57% (8/14 amplicons) of the synthetic control genome were able to be sequenced up to ∼*C_T_* 32, after which no amplicons were produced. Regions which were not amplified at higher viral loads were A2, A3, A4, B4, B5 and B6, signaling that that these primer pairs span two contiguous but separate segments of the synthetic genome. The smaller tiled amplicons from ARTIC v3 produced a higher proportion (93.9%, 92/98 amplicons) of the genome; however, amplicons 16, 17, 33, 50, 66, and 82 were not amplified. Missing regions from both ARTIC v3 and long-amp methods overlapped, confirming six distinct segments of the synthetic control. Due to the nonamplification of larger products from the long-amplification method, less of the genome was able to be recovered, meaning that subsequent variant calling from these missing regions could not be performed. Complete genomes (>99% coverage) for the synthetic control was able to be obtained using RVOP up to a *C_T_* value of 28.

### Comparison of genome coverage across three sequencing methods.

**FIG 2:**
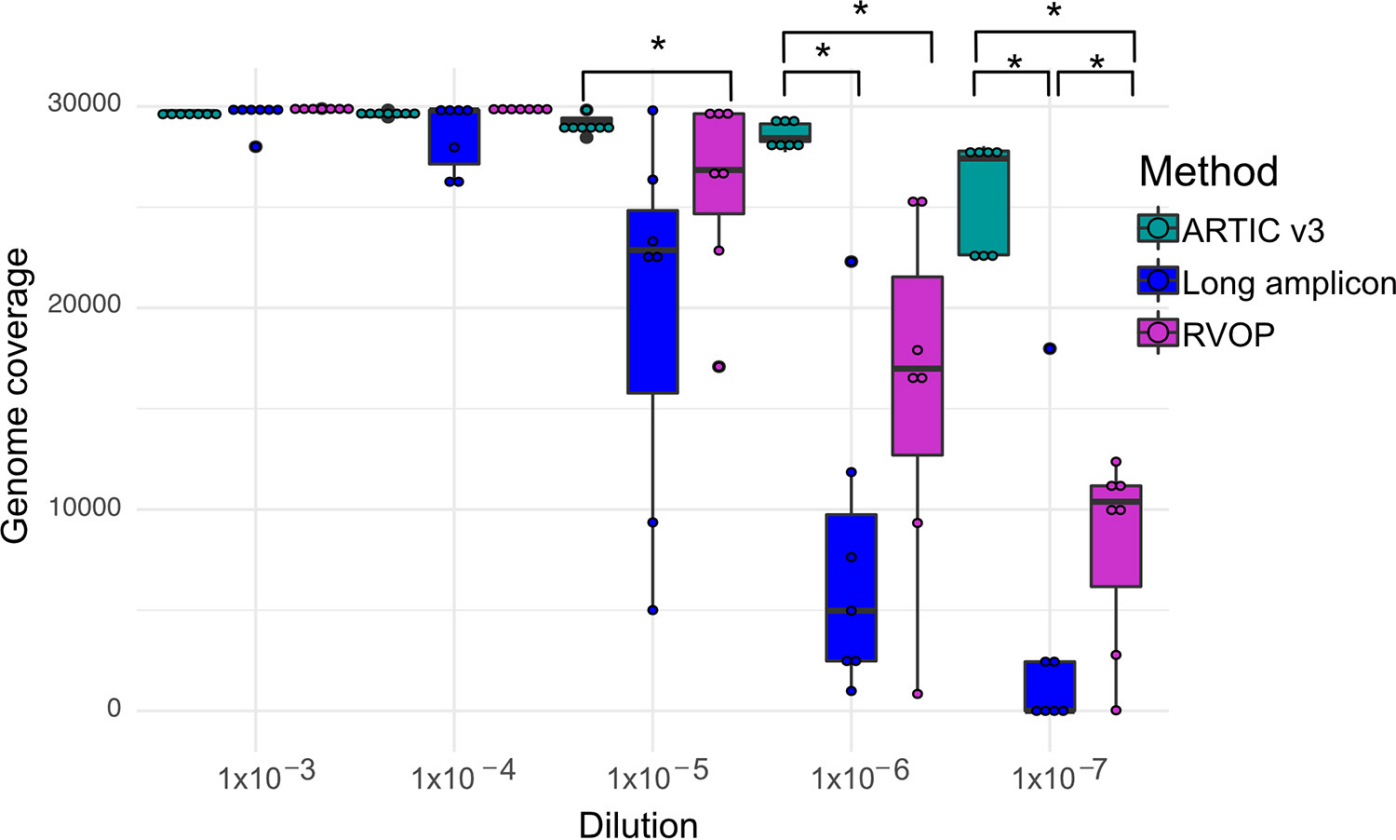
Box plot showing the SARS-CoV-2 genome coverage achieved by ARTIC v3-, long-amp-, and RVOP-based whole-genome sequencing methods of SARS-CoV-2 performed on serial dilutions of SARS-CoV-2 cultures. The bold black line within the box plot represents the median coverage, the box represents the interquartile range, and the whiskers denote the range of median coverage at each dilution. Significant differences were observed in genome coverage between different methods (*, *P* < 0.05). Pairwise comparisons between methods were performed only within each dilution.

Median raw read counts obtained from dilutions prepared using ARTIC v3 (1,217,844; range, 242,390 to 1,776,118) and RVOP (1,260,356; range, 528,334 to 6,972,838) were comparable; however, lower raw read counts were obtained using long-amp (532,512; range, 118,528 to 970,614) where the number of total mapped reads decreased with SARS-CoV-2 viral load (Fig. S1 [supplemental files 1 and 6]). At *C_T_* values of 25 to 29 (up to 2,000 copies/μl), all three WGS methods generated nearly complete SARS-CoV-2 genomes with >10× coverage (Fig. 2; Fig. S1 [supplemental files 1 and 6]). The highest level of genome coverage across all five dilutions was achieved using ARTIC v3, with >90% genome coverage achieved at viral loads down to a *C_T_* value of ∼38 (2 copies/μl). For each of the complete genomes (expected genome size of 29,903 bp), there were fewer than 1,000 ambiguous bases (Ns) from the reference genome (GenBank accession no. MN908947.3) (Fig. 2). On the other hand, genome coverage decreased substantially using long-amp and RVOP methods at a median *C_T_* of 32 (range, 30.7 to 33.4; median viral load, 1,340 copies/μl; range, 725 to 14,613 copies/μl) (Fig. S1 [supplemental files 1 and 6]); however, the differences observed were not significant (Fig. 2). This decreasing trend continued at lower dilutions for both long-amp and RVOP, resulting in significant differences of the genome coverage obtained using the ARTIC v3, long-amp, and RVOP methods (*P* < 0.05) (Fig. 2).

### Read depth affects genome coverage and variant calling.

**FIG 3:**
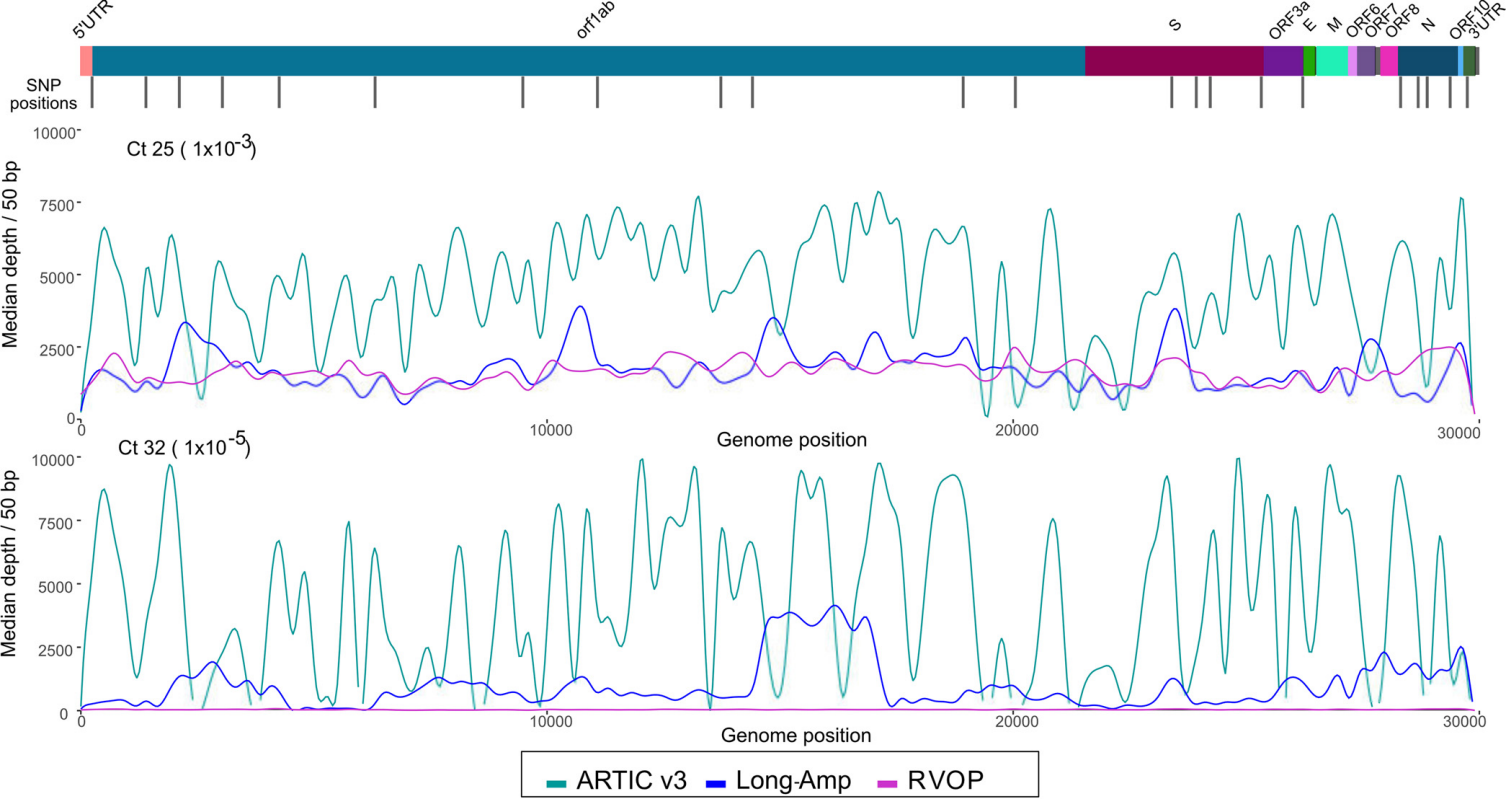
Overall read depth across the SARS-CoV-2 genome using ARTIC v3 (green line), long-amp (blue line), and probe capture RVOP (pink line) whole-genome sequencing methods. Depth was averaged across all samples for each method separately. Lines were smoothed by using the geom_spline function in R. The colored bar at the top represents the regions of the SARS-CoV-2 genome, and black bars represent informative single nucleotide polymorphisms.

Read depth across amplicons differed substantially between the ARTIC v3 and long-amp methods, creating highly uneven genome coverage. ARTIC v3 amplicons 9, 17, 23, 64, 67, 70, 74, and 91 were amplified inconsistently at higher *C_T_* values (*C_T_* > 34). A2, B3, and B6 from the long-amp protocol were the poorest performing, often not amplified in samples with a *C_T_* of <30. These 400-bp to 5-kb missing amplicons created large genomic gaps, which made variant calling problematic. In contrast, the amplicons which amplified with high efficiency using ARTIC v3 (amplicons 44, 57, and 62) had consistently higher average read depths regardless of *C_T_* value. The RVOP achieved the most consistent read depth across the genome, with relatively even distribution of missing bases compared with either amplification-based sequencing method. However, average read depth of samples at a *C_T_* of ∼32 (range, 30.7 to 33.4) was low (Fig. 3), with inconsistent genome coverage of <10×, also resulting in problems with variant calling.

### ARTIC rebalanced pools.

**FIG 4:**
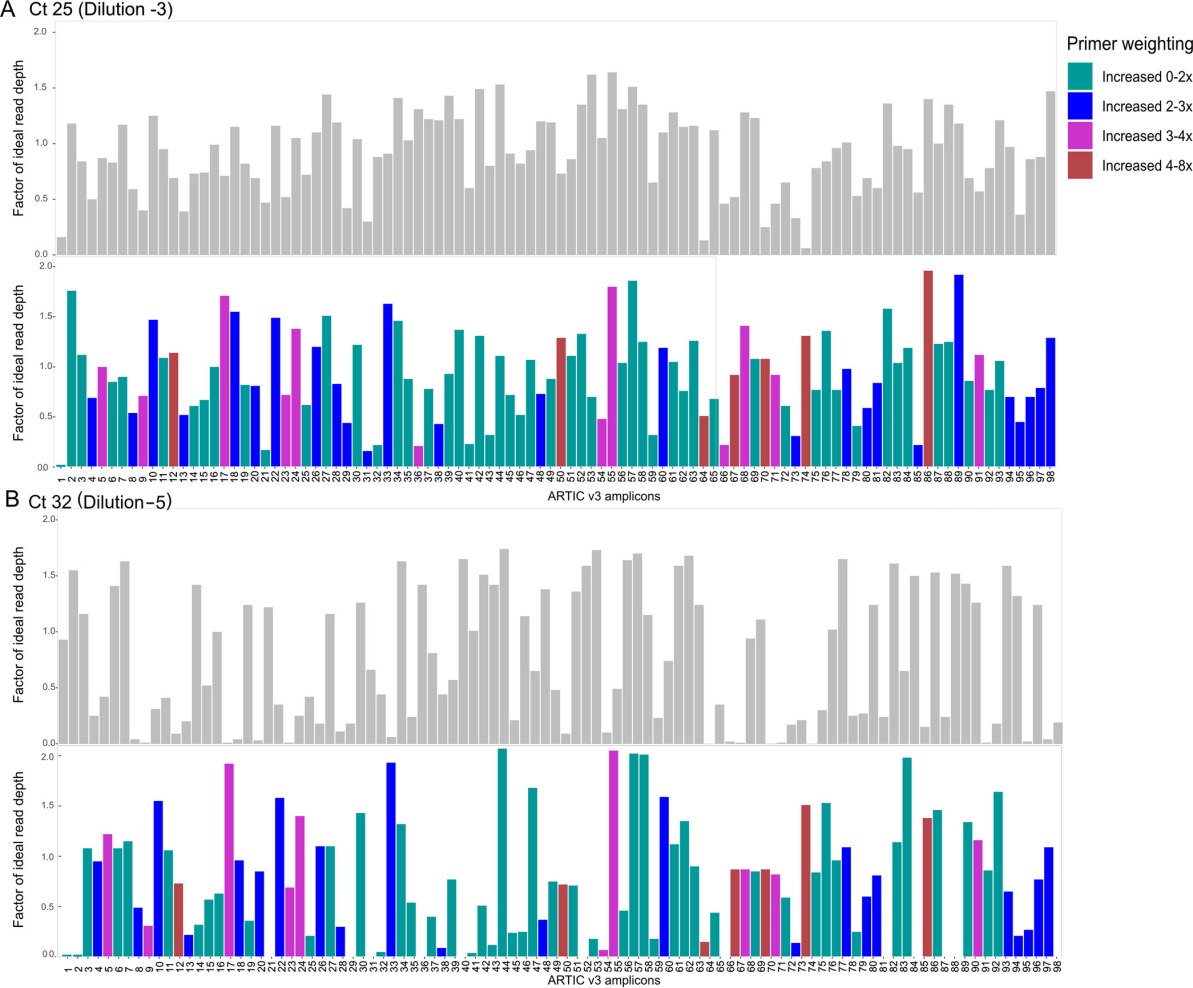
Comparison between ARTIC v3 original primer pooling and rebalanced primer pools at two dilutions (1 × 10^−3^ [*C_T_*, 25] and 1 × 10^−5^ [*C_T_*, 32]). Median read depth per ARTIC v3 amplicon was assessed in nonoverlapping segments. This median depth per amplicon was then converted to a factor of the expected read coverage (total mapped reads/genome size × 150 bp). The resulting depth factor is indicative of an underrepresented amplicon if the ratio is <1 and an overrepresented amplicon if the ratio is >1. These factors were compared between original and rebalanced ARTIC v3 sequencing runs. Gray bars represent the factor of sequencing depth achieved by the standard ARTIC v3 pooling protocol, whereas colored bars represent sequencing depths of rebalanced ARTIC v3 primer pools. ARTIC v3 primers are listed across the *x* axis in sequential order across the genome. Bar colors indicate primer weightings or the additional concentration of primer added in the rebalanced primer pools. For exact primer weightings, refer to https://www.protocols.io/view/covid-19-artic-v3-illumina-library-construction-an-bibtkann.

Using the COVID-19 Genomics Consortium (COG-UK) guidelines, we rebalanced ARTIC v3 primers in an attempt to improve amplification of specific amplicons and obtain more even sequencing coverage across the genome. Figure 4 shows the performance of rebalanced primers compared with original primer concentrations prior to rebalancing. Unsurprisingly, as viral load decreased, coverage across more poorly performing amplicons decreased in parallel (Fig. 4; Fig. S1 [supplemental files 1 and 6]). No significant changes in coverage were observed (across all dilutions) with amplicons 15, 27, and 73, even though the primer concentrations were increased 1.5× to 2.1×. However, amplicons 64, 67, 70, and 74 (for which primer concentrations were increased by a factor of 6 to 7.8) performed significantly better than original unbalanced primer pools. Other amplicons (i.e., 36, 54, and 66) whose primers were increased by a factor of >3 performed worse than expected. Regardless of individual primer rebalancing factors, sufficient depth (>10×) to meet variant calling QC at a *C_T_* of 35 was obtained for all amplicons.

### Comparative sensitivity of three SARS-CoV2 sequencing methods.

**FIG 5:**
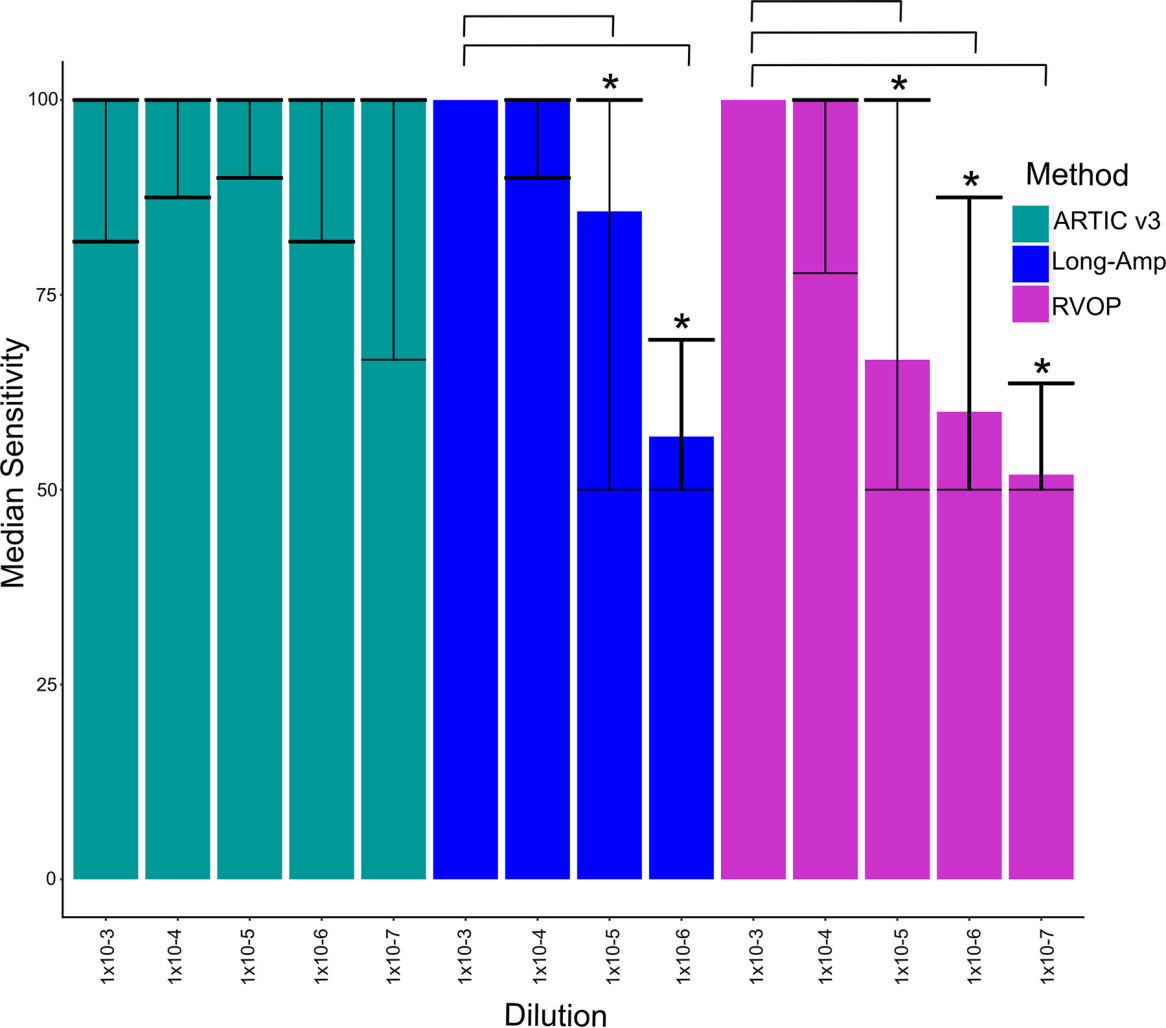
Sensitivity of ARTIC v3, long-amp, and RVOP whole-genome sequencing methods. The sensitivity of ARTIC v3 was the highest across all viral load dilutions. No sensitivity calculations could be made for the pooled long-amplification method at 10^−7^ dilutions due to insufficient amplicons for variant calling. Significant differences (*) in sensitivity were observed between the 10^−3^ dilution and the 10^−5^ and 10^−6^ dilutions using the long-amp method and between the 10^−3^ dilution and the 10^−5^, 10^−6^, and 10^−7^ dilutions using the RVOP method. No differences were observed between ARTIC v3 original primer pooling and rebalanced pools.

Sensitivity of each method was defined as the ability to accurately call SNPs, based on a clear consensus among all the dilutions. All three methods exceeded 90% sensitivity with a median *C_T_* of 28.7 (range, 27.6 to 31.3; median viral load, 12,025 copies/μl) (Fig. 5). The sensitivity for ARTIC remained high for samples up to a *C_T_* of >38, whereas sensitivities for both pooled long-amp and RVOP dropped below 80% at a *C_T_* of >30. Specificity was high, ranging between 100% and 99.97% across all methods and dilutions. False SNP detections ranged from 0 to 3 SNPs per genome (long-amp: median, 0; range, 0 to 1; ARTIC v3: median, 0; range, 0 to 2; RVOP: median, 1; range, 0 to 3) and were more common at dilutions of 1 × 10^−6^ to 1 × 10^−7^ (14/15 false-positive SNPs). No differences in sensitivity or specificity were observed between ARTIC v3 original primer pools and rebalanced primer pools.

### RVOP and the detection of other respiratory pathogens.

The RVOP can detect 43 common human respiratory viruses and 60 human control genes (which serve as internal positive controls for library construction and sequencing steps) in individual clinical samples (28). Trimmed reads from all seven diluted cultures prepared using the RVOP were mapped against 203 reference sequences of 43 respiratory pathogens. Human rhinovirus 89 (NC_001617.1) and adenovirus C (NC_001405.1) were detected, although coverage across both viral genomes was less than 2%. An in-house respiratory panel RT-PCR confirmed the presence of both rhinovirus (*C_T_*, 27) and adenovirus (*C_T_*, 26) in the SARS-CoV-2-negative respiratory matrix (Table S3 [supplemental file 5]). Twenty-seven reads mapped to human coronavirus 229E, but when BLAST was used to check the identity of these reads, the majority of mismapped reads also had high homology with SARS-CoV-2 and were subsequently found to have short read lengths (<40 bp).

### Low-frequency-variant detection.

A synthetic SARS-CoV-2 construct was used to control for low-frequency variants which arise due to artifacts of the amplification, enrichment, or sequencing process. With the RVOP method, 12 low-frequency variants were detected, 8 of which were replicated in two or more serial dilutions or by two different methods. When the synthetic control was used, the long-amp method generated five low-frequency variants, two of which were replicated at the same genomic position in two or more dilutions. The ARTIC v3 method generated only two low-frequency variants, and only one genomic position was reproduced in serial dilutions (Fig. S2 [supplemental files 2 and 3] and 3; Table S2 [supplemental file 5]). The synthetic control was also serially diluted in RNase-free water to identify any low-frequency variants derived from the negative respiratory matrix. The profile of variants detected using ARTIC and long-amp in water was smaller with four and two variants, respectively. However, two attempts failed to produce libraries using the RVOP method, likely due to the low-biomass input when the SARS-CoV-2 synthetic control was diluted in water.

Within the spiked cultured specimens, an average of 16.7 low-frequency variants were detected using all three techniques per sample (range, 12 to 25). However, almost half of these low-frequency variants were removed, due to their detection in a single dilution per isolate. Generally, these nonreplicated low-frequency variants were detected only in low-viral-load dilutions (1 × 10^−6^ and 1 × 10^−7^). Low-frequency variants repeatedly detected in at least two dilutions were most commonly detected using RVOP (median number of sites, 10; range, 6 to 16) followed by ARTIC v3 (median number of sites, 1; range, 0 to 5) and long-amp (median number of sites, 1; range, 0 to 4) (Fig. S2 [supplemental files 2 and 3]; Table S2 [supplemental file 5]). The presence of low-frequency variants was confirmed, at the same genome position by all three methods, in two culture isolates (median number of sites, 2; range, 0 to 4): isolate 1 at positions 657, 27972, and 29585 and isolate 2 at positions 12299 and 16466 (Table S2 [supplemental file 5]). When comparing the genomic position of low-frequency variants detected in the synthetic control and the spiked respiratory matrix, we uncovered five variants that were in the same genomic positions of all seven spiked specimens and the synthetic control, indicating that the RVOP produces artifactual signal (Table S2 [highlighted in bold; supplemental file 5]; Fig. S2 [supplemental files 2 and 3] and S3 [supplemental files 4 and 6]). No additional low-frequency variants were detected using the ARTIC v3 rebalanced pools. Despite using a simulated respiratory matrix to control for background artifacts, there was little consistency in the number and location of low-frequency variants detected across the diluted genomes using each of the three methods.

## DISCUSSION

This study highlights important quality requirements for high-throughput sequencing of SARS-CoV-2 for the purpose of public health surveillance. These parameters are critical for the application of SARS-CoV-2 genomics in tracking transmission pathways and monitoring ongoing viral evolution in circulating virus populations. Sequencing of samples with low viral loads and high *C_T_* values (e.g., >33) has been challenging regardless of the methodology used (29–32). Sequencing of such samples can still be attempted, but the resulting genomes often have a substantial portion of missing bases, making it difficult to infer genomic clusters or identify VOC.

Our findings demonstrated the rapid loss of genome coverage using pooled long-amp sequencing and the RVOP at a *C_T_* of >32 (median viral load, 1,340 copies/μl), indicating that low viral load or suboptimal RNA quality can be a limiting factor that must be considered when these methods are used to generate reproducible genomic data. In contrast, nearly complete genomes can be recovered using ARTIC v3 at a *C_T_* of >38, suggesting that the ARTIC protocol is either more sensitive at low viral loads or less impacted by reduced RNA quality. Indeed, the ARTIC protocol has performed well for samples with higher viral loads (*C_T_* < 25) (33–35) and has been implemented in numerous laboratories worldwide. However, at lower viral loads, we found that both amplification-based methods inconsistently produced data in genomic regions of known significance. Analogous to the findings presented here, uneven amplification efficiencies and coverage bias have been widely reported for low-viral-load specimens (34, 35). Increasing coverage over underperforming regions of the genome may be achieved by sequencing at greater depths, but this approach is costly and impractical in outbreak situations where high and rapid throughput is necessary. Rebalancing primer concentrations for ARTIC v3 improved coverage over previously poorly sequenced regions, and it is likely that additional manipulation of primer pooling or primer design would further enhance coverage.

In contrast, the RVOP generates consistent and even SARS-CoV-2 genome coverage over a range of *C_T_* values, despite the sensitivity being only marginally higher than for long-amp sequencing. While not examined fully in this study, the RVOP can simultaneously detect other pathogens in a single sample, reducing delays in diagnosis and treatment options for patients who test negative for SARS-CoV-2. Similar to the genome coverage achieved for SARS-CoV-2 in this study, the RVOP should also be able to generate whole genomes of other respiratory viral pathogens targeted by the panel. We were unable to confirm complete coverage of adenovirus and rhinovirus (despite their presence being confirmed by RT-PCR), as the pooled respiratory matrix used for this study consisted of a convenient sample of SARS-CoV-2-negative universal transport medium (UTM). Poor sample quality as a result of suboptimal transport and storage conditions may have been another factor contributing to the limited and inconsistent coverage of other respiratory pathogens.

The loss of informative sequencing data, especially in genomic regions of interest, can hamper public health efforts to monitor changes in circulating viral populations. Given that numerous VOC have been identified worldwide (17, 36–38), amplicon dropouts, particularly within the spike region, are problematic. For instance, B6 from the long-amp protocol and amplicons 70 and 74 from the original ARTIC v3 protocol encompass part of the spike protein, but all performed poorly and often were not amplified at a *C_T_* of >32. Rebalancing the ARTIC v3 primer pools increased sequencing coverage and depth over amplicons 70 and 74. However, it is important to note that both long-amp and ARTIC v3 methods involve primer binding prior to amplification and are therefore prone to amplicon dropouts if variants arise within primer sites. The risk of amplicon dropouts can be overcome by redesigning primers away from variant sites; such protocol changes can be time-consuming and difficult to implement but will be necessary given the rapid rise and spread of VOC. The constantly changing population dynamics of the circulating SARS-CoV-2 viruses will require ongoing, high-quality genomic surveillance to track the evolution of circulating isolates and help inform necessary changes to sequencing methodologies.

Detecting and locating genomic positions of low-frequency variants from culture-derived specimens can provide insight into the reliability of intrahost single nucleotide variants (iSNVs) called from clinical specimens. The role of intrahost genomic variability in SARS-CoV-2 may be important in inferring transmission events (39) and may be responsible for significant complications in patients with malignancies (40, 41). Thus, such low-frequency variants require ongoing detection and surveillance. There have been suggestions that iSNVs can be detected at a frequency as low as 2%; however, only iSNVs occurring at a frequency of >10% and a minimum coverage of 100× were investigated in the present study. At this threshold, substantial variability of low-frequency variants was observed using the methods tested in this study even after controlling for background artifacts generated during the WGS process (via the use of viral cultures in a defined respiratory matrix and a synthetically produced viral construct). The RVOP method detected the highest number of low-frequency variants; however, five variants at the same genomic location were detected in each of the spiked isolates and also in the synthetic control, suggesting that these variants might be an artifact of the RVOP method. In general, low-frequency variants were inconsistently detected in the same specimen using different methods. This inconsistency can be attributed to the unique sequencing chemistries of each method and to the impact of upstream amplification and hybridization procedures, highlighting the importance of recognizing and accounting for biases that arise during both laboratory preparation and downstream bioinformatic processes.

While we have systematically tested and determined the threshold at which complete genomes can be generated for each method, we have not yet addressed issues with poor-quality specimens. Quality and quantities of RNA in clinical specimens for WGS are highly dependent on sample types, collection methods, transport, and processing. Suboptimal processes are not uncommon and are inherent in high-throughput and often centralized testing. Sample degradation as a result of these factors has been highlighted as a significant problem in generating high-quality genome sequences (32).

In conclusion, our systematic evaluation of sensitivity and ability to detect low-frequency variants demonstrated that overall, the ARTIC v3 protocol was the most sensitive and cost-effective method for generating complete SARS-CoV-2 genomes. The additional advantages of the ARTIC protocol are better capacity to recover genomes from clinical samples with low viral loads and the ability to detect low-frequency variants. Ongoing updates to the ARTIC v3 protocol, such as the rebalancing of primer pools (through the COG-UK and efforts from research institutions), will ensure continual improvements to the WGS process. The optimization of SARS-CoV-2 genome sequencing can increase the utility of SARS-CoV-2 genomics for COVID-19 cluster detection, transmission tracking, and public health responses.
