# Supplementary material for: SARS-CoV-2 Genome Sequencing Methods Differ in Their Abilities To Detect Variants from Low-Viral-Load Samples
Source: J Clin Microbiol. 2021 Oct 19;59(11):e01046-21. doi: 10.1128/JCM.01046-21 (PMC8525559; doi:10.1128/JCM.01046-21)
Supplement: Supplemental file 5 — Tables S1, S2, S3, and S5 and caption of Table S4. Download JCM.01046-21-s0006.pdf, PDF file, 0.1 MB [file jcm.01046-21-s0006.pdf]

SARS-CoV-2 GENOME SEQUENCING METHODS DIFFER IN THEIR ABILITY TO DETECT VARIANTS FROM LOW VIRAL LOAD SAMPLES

C. Lam[1], K. Gray[1,2], M. Gall[2], R. Sadsad[1,3,4], A. Arnott[2], J. Johnson-Mackinnon[1,3], W. Fong[1], K. Basile[2,3], J. Kok[1,2], D. E. Dwyer[1,2,3] V. Sintchenko[1,2,3], R.J. Rockett[1,3]

Supplementary Materials

Supplementary Table 1: Genomes of SARS-CoV-2 isolates used in the study

Isolate ID	GISAID ID	Lineage	SNP profile
Isolate 1 (NSW05)	EPI_ISL_412975	B.4	G:1397:A, G:4255:A, *G:11083:T*, A:20047:G, T:28688:C, G:29742:T
Isolate 2 (NSW13)	EPI_ISL_413599	B.4	G:1397:A, C:2113:T, G:11083:T, C:18928:T, C:26213:T*, T:28688:C, G:29374:A, G:29742:T
Isolate 3 (NSW08)	EPI_ISL_413594	B	G:25323:T
Isolate 4 (NSW14)	EPI_ISL_413600	B.4	G:1397:A, G:4255:A, *G:11083:T*, A:20047:G, T:28688:C, G:29742:T
Isolate 5 (NSW155)	EPI_ISL_427647	B.1	C:241:T, C:3037:T, C:14408:T, A:23403:G, G:28881:A, G:28882:A, G:28883:C
Isolate 6 (NSW26)	EPI_ISL_417389	B.4	G:1397:A, C:2113:T, A:9483:G, *G:11083:T*, C:18928:T, G:24227:A, T:28688:C, G:29374:A, G:29742:T
Isolate 7 (NSW48)	EPI_ISL_417403	B.6	C:6312:A, *G:11083:T*, C:13730:T* , C:23929:T, C:28311:T

The * symbol denotes single nucleotide polymorphisms (SNPs) lost in culture. The mutation lost in Isolate 2 was detected as a low frequency variant. The SNP lost during culture of Isolate 7 was not detected as a low frequency variant. Italics indicate known hypervariable sites.

Supplementary Table 2. Low frequency variants detected using each method

**Sample ID**	**Long-Amp**	**ARTIC v3**	**RVOP**
TWIST Control -UTM	3350, 6669, *11074(+T)*, 11079	*11074(+T),* 14707, 26791, 26793, 26794, 26796	**5765**, **5766**, 10001, *11074(+T)*, **12413**, **12926** (+C), **23652**, **26433**, 26791, 26793, 26794
TWIST Control -Water	3350, 6669	26791, 26793, 26794, 26796	N/A
Isolate 1.	657, *11082(-N)*, 27972, 29585	657, *11082(-N)*, 27972, 29585	657, **5765**, **5766**, *11082(-N)*, **12413**, **12926 (+C)**, 15071, 17561, 18408, 18848, 20079, 23403, **23652**, **26433**, 27870, 27972, 29585
Isolate 2.	*10323*, *11074(+T)*,*11082(-N)*, 12299, 16466	*10323*, *11082(-N)*, 12299, 16466	**5765**, **5766**, *11082(-N)*, 12299, 16466, 18402, 21055, 21949, **23652**
Isolate 3.	-	-	**5765**, **5766**, **12413**, **12926 (+C)**, **23652**, **26433**, 27870
Isolate 4.	*11082(-N)*	*11074(+T)*, *11082*	**5765**, **5766**, *11082(-N)*, **12413**, **12926** (+C), 17561, 20079, 23403, **23652**, **26433**, 27870
Isolate 5.	695, 29190	-	**5765, 5766, 12413, 12926, 23652**, 27870
Isolate 6.	11050, *11082(-N)*	*11082(-N)*	**5765, 5766**, *11082(-N)*, **12413**, **12926** (+C), 15071, 19812, 21949, **23652**, **26433**, 27870
Isolate 7.	6310, *11074(+T)*,	*11082(-N)*	**5765**, **5766**, 6310, *11082(-N)*, **12413**, **12926** (+C), 15071, **23652, 26433**, 27870

Low frequency variant positions are based on the reference SARS-CoV-2 genome (NCBI GenBank accession MN908947.3). Underlined and italic positions are known regions of

hypervariability commonly masked from phylogenetic analysis, indels are denoted with brackets indicating the inserted base or N for a deleted base. Bold positions indicate intra-host single nucleotide variants (iSNV) sites which occurred in both the TWIST control and spiked isolate RNA, indicative that the iSNV is likely an artefact and may occur from non-specific mapping of non-SARS-CoV-2 RNA.

Supplementary Table 3:

Negative SARS-CoV-2 RNA extract and detection of other respiratory pathogens

**Respiratory pathogen**	**Ct Value**
Rhinovirus	27
Adenovirus	26
Influenza	ND
RSV	ND
Parainfluenza	ND

Key: ND – not detected; RSV - Respiratory syncytial virus

Supplementary Table 4: Details of SRA data availability for all 118 genomes produced in the study (external spreadsheet)

Supplementary Table 5: Details of hands-on-time and reagent cost of each methodology

**Required prep time**	**ARTIC v3**	**Long Amp**	**RVOP**
Hands on time (excl seq run)	9 hr 40 min	11hr 5 min	7 to 7.5 hr
Sequencing time required	12hrs	12hrs	12hrs
Total time (incl sequencing run)	21hr 40min	23hr 5 min	19hr to 19.5hr
Cost (Extraction, PCR, Library Preparation and 75bp Sequencing)	AUD$122	AUD$163	AUD$238
