# Supplementary material for: SARS-CoV-2 Genome Sequencing Methods Differ in Their Abilities To Detect Variants from Low-Viral-Load Samples
Source: J Clin Microbiol. 2021 Oct 19;59(11):e01046-21. doi: 10.1128/JCM.01046-21 (PMC8525559; doi:10.1128/JCM.01046-21)
Supplement: Supplemental file 6 — Legends of Fig. S1 to S3. Download JCM.01046-21-s0007.pdf, PDF file, 0.06 MB [file jcm.01046-21-s0007.pdf]

Supplementary Figure Legends:

Supplementary Figure 1

Quantification of median raw and mapped reads for ARTIC v3 (green), Long Amp (blue) and RVOP (pink) at each dilution. Libraries were pooled for sequencing with the aim of generating $1x10^6$ reads per specimen. Median raw read counts ranged between $4x10^5$ and $1.6x10^6$. The number of mapped reads decreased over serial dilutions of SARS-CoV-2 cultures. Median counts are represented by bold black lines in the centre of each box at serial dilutions, lower and upper limits of the box represent the interquartile range and whiskers represent the minimum and maximum counts. The asterisks (*) denotes a single library which was excluded from Figure 1. This library (NSW13, dilution 1x10-7, RVOP) generated $7x10^6$ raw reads with only 1006 reads mapping to SARS-CoV-2.

Supplementary Figure 2

Comparison of read depth for Long-amp method (blue), ARTIC v3 (green) and the respiratory viral oligo panel (RVOP- pink) at Ct 25 (panel A) and Ct 32 (panel B). The depth of coverage is shown on the right axis, while the proportion of reads for single nucleotide polymorphisms (SNPs) is on the left axis. The position of SNPs (red circles) and low frequency variants (orange circles, detected in >=2 dilutions per specimen) across the genome are overlaid on top of both coverage graphs.

Supplementary Figure 3

Low Frequency variants detected in synthetically generated SARS-CoV-2 RNA using three methodologies; (ARTIC v3, Long-amp and RVOP). These low frequency variants are detected in more than one dilution when five serial dilutions of a synthetic construct of wild-type Wuhan-1 SARS-CoV-2 RNA are sequenced. The variants are considered artefacts of the viral enrichment, amplification or sequencing process as they should not be contained within the synthetic RNA control. Some variant positions could be reliably detected in a sub-population of mapped reads when the synthetic control was diluted in water or SARS-CoV-2 negative respiratory RNA matrix. The top panel of graphs depict variants detected by the long-amp methodology in SARS-CoV-2 negative respiratory matrix and water. The middle panel demonstrates variants detected by the ARTIC v3 methodology in SARS-CoV-2 negative respiratory matrix and water. The lower graph depicts variants detected using the RVOP, after two attempts a library could not be prepared when diluting the synthetic control in water,

thought to be due to the low biomass in the sample. Pink and purple shaded regions indicate regions of the genome that were unable to be amplified, due to the five non-overlapping segments of RNA.
